# Can increasing footwear bending stiffness ameliorate age-related mechanical and metabolic deficits in walking?

**DOI:** 10.7717/peerj.21563

**Published:** 2026-07-30

**Authors:** Daniel J. Davis, Christopher L. Long, Jason R. Franz, Kota Z. Takahashi

**Affiliations:** 1 Department of Health & Kinesiology, University of Utah, Salt Lake City, UT, United States of America; 2 Lampe Joint Department of Biomedical Engineering, University of North Carolina at Chapel Hill and North Carolina State University, Chapel Hill, NC, United States of America; 3 Department of Biomedical Engineering, University of Utah, Salt Lake City, UT, United States of America; 4 Department of Physical Medicine & Rehabilitation, University of Utah, Salt Lake City, UT, United States of America

**Keywords:** Carbon fiber insoles, Ankle joint moment, Distal to proximal shift, Older adults, Assistive devices, Footwear technology, Metabolic cost of walking, Sloped walking

## Abstract

Older adults consume metabolic energy faster than their younger adult counterparts while walking, particularly on sloped terrain. This increased rate is likely in part due to an age-related shift in moment and power production from the ankle joint to the hip. Shifting these mechanics proximally may occur due to older adults losing more mechanical energy at the foot and producing lower ankle joint moments, deficits that are exaggerated when walking uphill or against impeding forces. A promising method to target some of these foot and ankle mechanics differences is increasing footwear longitudinal bending stiffness via carbon fiber insoles. We examined how increasing footwear stiffness alters older adult (9F/10M, 70 ± 5.9 yrs) walking energetics across slopes. Increasing footwear stiffness reduced the magnitude of negative power from the foot+shoe structures distal to the forefoot, but increased negative power of the foot+shoe structures distal to the hindfoot (foot+shoe complex as a whole). At the ankle, peak joint moment increased by ∼10% in the stiffest footwear compared with the baseline standardized shoe. Neither knee and hip mechanics nor net metabolic power were consistently affected by footwear stiffness. These results indicate that increasing older adult footwear stiffness via flat carbon fiber insoles attenuates some age-related deficits in foot and ankle mechanics, but does not result in a clear attenuation of the age-related distal to proximal shift in joint mechanics or clear metabolic benefits. The increase in ankle joint moment with stiffer footwear could be useful longer-term as a means to strengthen the calf musculature of older adults. Future work should examine the effects of footwear stiffening on ankle muscle-level outcomes such as force production, excitation, and fascicle mechanics.

## Introduction

Older adults consume metabolic energy at a faster rate than younger adults while walking (Das Gupta, Bobbert & Kistemaker, 2019; Peterson & Martin, 2010). This greater metabolic demand is thought to contribute to older adults’ greater fatigue, reduced physical activity, and diminished walking speed; together associated with all-cause mortality (Egerton et al., 2016; Schrack et al., 2016; Studenski et al., 2011). As such, interventions aimed at reducing the metabolic cost of walking for our growing population of older adults are needed.

Analyses and interventions targeting the function of structures spanning older adults’ ankle joints have received considerable attention (*e.g.*, Browne & Franz, 2019; Lakmazaheri et al., 2024). This is because increased metabolic energy expenditure in older adults has been proposed to arise in no small part from changes that occur at the level of the ankle joint and its musculature. Specifically, these deficits at the ankle are thought to contribute to the shift of moment production and energy generation from the ankle joint musculature to the hip joint musculature (Cofré et al., 2011; DeVita & Hortobagyi, 2000; Silder, Heiderscheit & Thelen, 2008). Recent evidence demonstrates that the muscle–tendon units of the ankle joint can indeed produce moments more efficiently than those crossing the hip joint (Fallah & Beck, 2025). Therefore, relying on less metabolically efficient hip musculature due to suboptimal ankle muscle function likely contributes to metabolically inefficient gait.

Ankle-specific deficits in older adults appear to be a combination of differences at both the ankle and the foot. Age-related foot muscle weakness has been widely reported (Endo, Ashton-Miller & Alexander, 2002; Menz et al., 2006; Mickle et al., 2016; Suwa et al., 2017), and older adults also generate less energy and lose more energy at the foot than younger adults (Krupenevich et al., 2021). These two phenomena may be linked, as Farris et al. (2019) demonstrated that inhibiting intrinsic foot muscle activation in younger adults results in a reduction in the mechanical energy generation at the foot. These findings highlight the need for interventions aiming to ameliorate older adult gait deficits to address functional differences not only at the ankle but also within the foot.

Carbon fiber insoles that increase footwear longitudinal bending stiffness alter both ankle joint and foot segment mechanics in younger adults (Davis et al., 2025; Ray & Takahashi, 2020; Takahashi et al., 2016). Specifically, increasing footwear stiffness increases ankle joint moments (Ray & Takahashi, 2020; Takahashi et al., 2016) and reduces mechanical energy lost by distal foot and shoe structures (primarily the metatarsophalangeal joint and midsole foam beneath the forefoot). Since older adults produce lower ankle joint moments than younger adults (DeVita & Hortobagyi, 2000; Franz & Kram, 2014; Silder, Heiderscheit & Thelen, 2008) and lose more energy at the foot (Krupenevich et al., 2021), these carbon fiber insoles may be well-suited for counteracting some characteristic age-related metabolic and mechanical energetic gait differences. Older adults also demonstrate reduced ankle joint mechanical power generation compared with their younger adult counterparts (DeVita & Hortobagyi, 2000; Franz & Kram, 2014; Silder, Heiderscheit & Thelen, 2008). In younger adults, increasing footwear stiffness slows ankle joint plantarflexion angular velocity, which can reduce ankle joint mechanical power generation as well (Ray & Takahashi, 2020). As such, increasing footwear stiffness allows for an examination of potentially offsetting effects of increased joint moment but decreased mechanical energy generation about the ankle in older adult walking.

Previously demonstrated effects of carbon fiber insoles may be even more pronounced when older adults walk on sloped terrain. Age-related increases in the rate of metabolic energy consumption are greater on slopes (Ortega & Farley, 2015). Older adults’ decrements in ankle joint moment production are also exaggerated when encountering uphill slopes (Franz & Kram, 2014; Waanders et al., 2019). At the foot, the magnitude of negative work absorbed by the structures distal to the forefoot’s center of mass is increased when younger adults walk uphill (Papachatzis & Takahashi, 2023). Age comparisons of foot energetics across slopes are missing, but Krupenevich et al. (2021) indicated that older adults demonstrate a greater magnitude of negative net mechanical work compared with younger adults when walking against impeding forces. Given the previously noted effects of carbon fiber insoles on ankle joint moment and energy lost by distal foot and shoe structures, these insoles may specifically target age-related metabolic and mechanical energetics differences that are exacerbated by sloped walking.

The purpose of this study was to examine how altering footwear bending stiffness *via* carbon fiber insoles influences older adult mechanical and metabolic energetics during gait tasks of varying demand (*i.e.,* different walking slopes). Our overarching hypothesis was that carbon fiber insoles would reduce older adults’ metabolic cost *via* coordinated changes to foot, ankle, and hip mechanics. We made several predictions pertaining to a plausible pathway for this to occur. First, predicted that increased footwear stiffness would increase older adults’ positive power and reduce the magnitude of negative power of distal foot structures. We also predicted that increased footwear stiffness would increase older adults’ peak ankle joint moment, and that this would result in concomitant reductions in peak hip joint moments and mechanical energy production. Lastly, due to these foot and lower-limb joint effects, we predicted that increased footwear stiffness would reduce older adults’ net metabolic power in a slope-specific fashion, with the magnitude of reduction aligning with the task demand (*i.e.,* a greater reduction in incline compared with level and decline walking).

## Materials & Methods

### Participants

An *a priori* power analysis was conducted in G*Power (v3.1.9.7; Faul et al., 2007) based on the effects of different footwear stiffnesses and walking speeds on younger adult whole-body metabolic cost and mechanical energy differences at the hip, knee, ankle, and foot (Ray & Takahashi, 2020). Our power analysis indicated that 17 participants were necessary to detect the smallest main or interaction effect of interest from Ray & Takahashi (2020) (positive knee work, partial eta squared (${\eta }_{p}^{2}$) effect size = 0.0526) at α = 0.05 and β = 0.8.

Nineteen (9F/10M, 70 ± 5.9 yrs, 73.8 ± 20.3 kg) older adults provided written informed) consent and participated in this IRB-approved study (University of Utah IRB #154429). Participants were screened for neural, cardiovascular, and musculoskeletal conditions that limited their ability to walk unassisted for at least six minutes. Additionally, participants were excluded if they had undergone lower-limb surgery in the past 12 months or were currently taking medication that could cause dizziness.

### Footwear

Participants wore standardized neutral footwear (New Balance 880 v13). Footwear stiffness was increased from the shoe only condition (hereafter referred to as the Low stiffness condition) by adding flat 1.6 mm (Medium stiffness condition) or 3.2 mm (High stiffness condition) thick carbon fiber insoles beneath the standard shoe insole. We have previously demonstrated that these insoles increase footwear longitudinal bending stiffness of a shoe approximately 4-fold and 12-fold for the two thicknesses, respectively (Ray & Takahashi, 2020; Takahashi et al., 2016). 3-point bending tests of the current footwear based on the protocol of Cigoja et al. (2019) indicated mean longitudinal bending stiffnesses of 12.9, 60.1, and 152.4 N/mm in the three conditions, in line with our previous estimates.

The different shoe conditions were not mass-matched, resulting in added masses between 29–42 g and 61–90 g per shoe in Medium and High stiffness conditions, depending on participant shoe size. For participants wearing the heaviest insole (High stiffness condition, men’s size 13 shoe), the added mass could be expected to result in a ∼1% increase in net metabolic power during level walking (Browning et al., 2007). Most participants wore smaller shoes than M13, therefore metabolic differences on average would be expected to be <1% during level walking. It is recognized, however, that the added mass may have a slightly greater effect on net metabolic power during incline and decline walking. We chose not to mass-match the conditions to prioritize ecological validity by benchmarking any translational benefit of using these specific insoles.

Participants were given at least five minutes to acclimate to each shoe condition prior to testing. A recent study indicated that at least 320 steps are required for older adults to acclimate to new athletic footwear (Soares et al., 2024). Based on common older adult step frequency (∼115 steps/min; Herssens et al., 2020), five minutes of walking allowed participants adequate time to achieve this 320-step minimum.

### Experimental protocol

**Figure 1:**
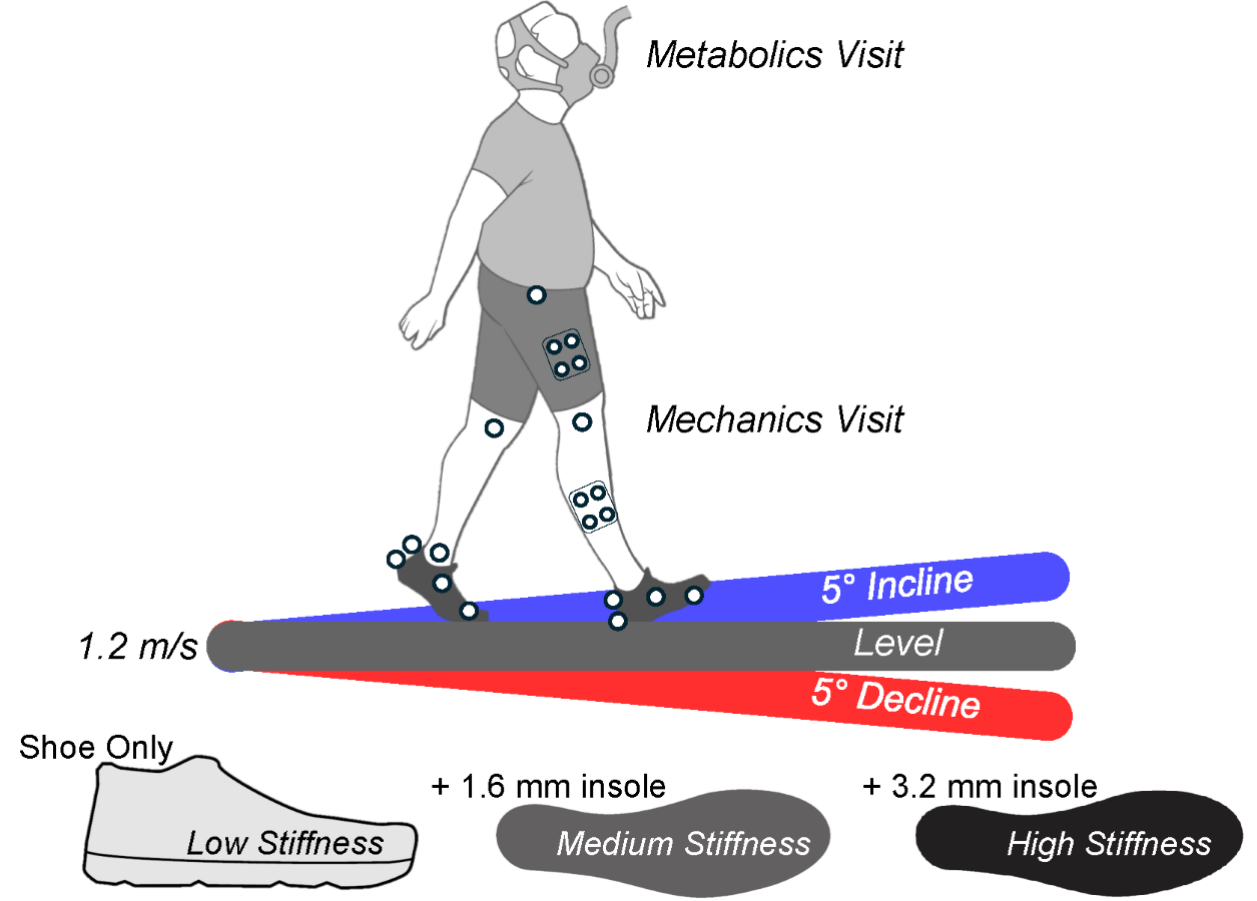
Experimental setup. Nineteen older adults walked at 1.2 m/s on a level, five-degree inclined, and five-degree declined force-instrumented treadmill. Participants walked in three different footwear conditions (Low, Medium, and High stiffness), wherein stiffness was increased by adding 1.6 mm or 3.2 mm flat carbon fiber insoles, respectively. During the “Mechanics Visit,” ground reaction force and marker position time-series were captured. A previously developed multi-segment foot model was used. During the “Metabolics Visit,” indirect calorimetry was performed. These visits were performed on separate days.

Similar to previous experiments analyzing mechanical and metabolic variables (*e.g.*, Beck, Schroeder & Sawicki (2004); Griffin, Roberts & Kram (2003); Ortega & Farley (2007)), participants completed two experimental visits (*metabolics visit* and *mechanics visit*) in a randomized order (Fig. 1). During both visits, participants walked at 1.2 m/s on an instrumented treadmill in nine different conditions: three footwear stiffness conditions (Low, Medium, and High) by three slope conditions (Level, 5° Incline, and 5° Decline). Together, these conditions allowed us to test both typical and more challenging walking conditions. Despite these challenges, our participants were able to walk for at least five minutes in all conditions with a respiratory exchange ratio ≤ 1.0. The order of the footwear and slope conditions were randomized separately for each participant. In other words, a given participant completed all three slope conditions in the first footwear condition before repeating that same slope order in the subsequent two footwear conditions.

During the *mechanics visit*, retroreflective markers were attached to the participant’s trunk, pelvis, thighs, shanks, and feet using a six degrees of freedom marker set that captured the movement of multiple foot segments (Bruening, Cooney & Buczek, 2012). Participants walked for at least 30 s at 1.2 m/s before ten seconds of data were captured. Marker positions were sampled at 250 Hz (Vicon, Oxford, UK) in synchrony with ground reaction forces sampled at 1,000 Hz (Bertec, Columbus, OH).

For the *metabolics visit*, participants arrived having abstained from caffeine and food for at least four hours and from exercise for at least two hours. Breath-by-breath rates of oxygen consumption and carbon dioxide production were measured using a metabolic cart (TrueMedics, Parvo, Sandy, UT) during five-minute standing rest trial and walking trials. Participants were given at least five minutes of rest between conditions.

### Data analysis

Mechanics data analyses were performed in Visual3D (HAS-Motion; Kingston, Ontario, Canada). Marker position and ground reaction force time-series were filtered at 6 Hz and 15 Hz, respectively, using a Butterworth filter applied in the forward and reverse directions. Participant-specific segment masses were estimated based on the regression equations of Dempster (1955). Body segment inertial properties were estimated based on Hanavan Jr (1964). Forefoot and hindfoot center of mass locations were estimated by modeling them as an elliptical cylinder and cylinder, respectively, of constant density according to the multi-segment foot model of Bruening, Cooney & Buczek (2012). Filtered marker position and ground reaction force time-series were used to estimate resultant joint moments expressed in the proximal segment reference frame *via* Newton-Euler inverse dynamics. We then computed six degrees of freedom joint power for the hip, knee, and ankle (Zelik, Takahashi & Sawicki, 2015). We modeled the ankle joint as the articulation between the tibia and hindfoot segment*s.* Previous analyses (Bruening, Cooney & Buczek, 2012; Zelik & Honert, 2018; Wager & Challis, 2024) have demonstrated that more traditional methods using a single segment foot model overestimate positive ankle joint power magnitudes and thus ankle joint power contributions. We deployed a ‘unified deformable’ analysis to quantify the power fluctuations of both biological foot and shoe structures distal to certain foot segments (Siegel, Kepple & Caldwell, 1996; Takahashi, Kepple & Stanhope, 2012). ‘Foot+Shoe Power Distal to the Hindfoot’ captures the energy fluctuations of all foot and shoe structures distal to the hindfoot center of mass (*i.e.,* the entire foot+shoe complex). ‘Foot+Shoe Power Distal to the Forefoot’ captures energy fluctuations distal to the forefoot center of mass, namely the metatarsophalangeal joint and midsole foam under the ball of the foot and toes.

Peak ankle plantarflexion moment during push-off, peak hip extension moment during early stance, and peak flexion moment during late stance were extracted. Joint and foot+shoe power time-series were time-integrated then divided by stride time to compute average mechanical power per stride. Peak resultant joint moments and average mechanical power were normalized with respect to body mass, then averaged across strides and between legs. Stride time was calculated using successive heel strike events, defined as the first sample at which the vertical ground reaction force exceeded 20 N. Steps in which one foot applied force to both force plates were excluded.

Metabolic data analyses were performed in MATLAB (v2023b; The MathWorks, Natick, MA, USA). Metabolic power in each condition was estimated from the gas exchange rates during the final two minutes of the *metabolics visit* trials using standardized equations (Kipp, Byrnes & Kram, 2018; Peronnet & Massicotte, 1991), then normalized with respect to body mass. The metabolic power during the standing rest trial was subtracted from the metabolic power during each walking trial to compute net metabolic power (W/kg).

### Statistical analysis

Average joint and foot+shoe mechanical power, peak ankle and hip moments, and net metabolic power between footwear and slope conditions were assessed using two-way within-subjects repeated measures analysis of variance (α = 0.05) in MATLAB. Normality was assessed using the D’Agostino-Pearson K^2^ test (D’Agostino & Pearson, 1973), and constant variance was assessed using Mauchly’s test. Non-parametric analysis of variance was employed if either the normality or constant variance test was statistically significant using a probability density function generated from 10,000 unique permutations of the data (Nichols & Holmes, 2002). In the presence of an interaction effect, *post-hoc* one-way repeated measures ANOVAs for the effect of footwear stiffness at each walking slope and the effect of slope at each footwear stiffness were conducted using the same parametric or non-parametric procedures. Effect sizes for each ANOVA factor were estimated using partial eta squared (${\eta }_{p}^{2}$) and Cohen’s *d* was used for *post-hoc* pairwise comparisons. Statistical significance for *post-hoc* comparisons was assessed using Holm-Bonferroni corrected *p*-values.

## Results

For brevity, only main effects of stiffness, slope × stiffness interaction effects, and *post-hoc* stiffness pairwise comparisons are presented here. Main effects and pairwise comparisons on the effect of slope are presented in File S1. Mechanical power variables at the foot and ankle joint for one participant were excluded due to marker tracking errors. One participant could not complete all nine stiffness and slope combinations during the *metabolics visit*, therefore their metabolic data were excluded.

### Foot+Shoe power distal to the forefoot

**Figure 2:**
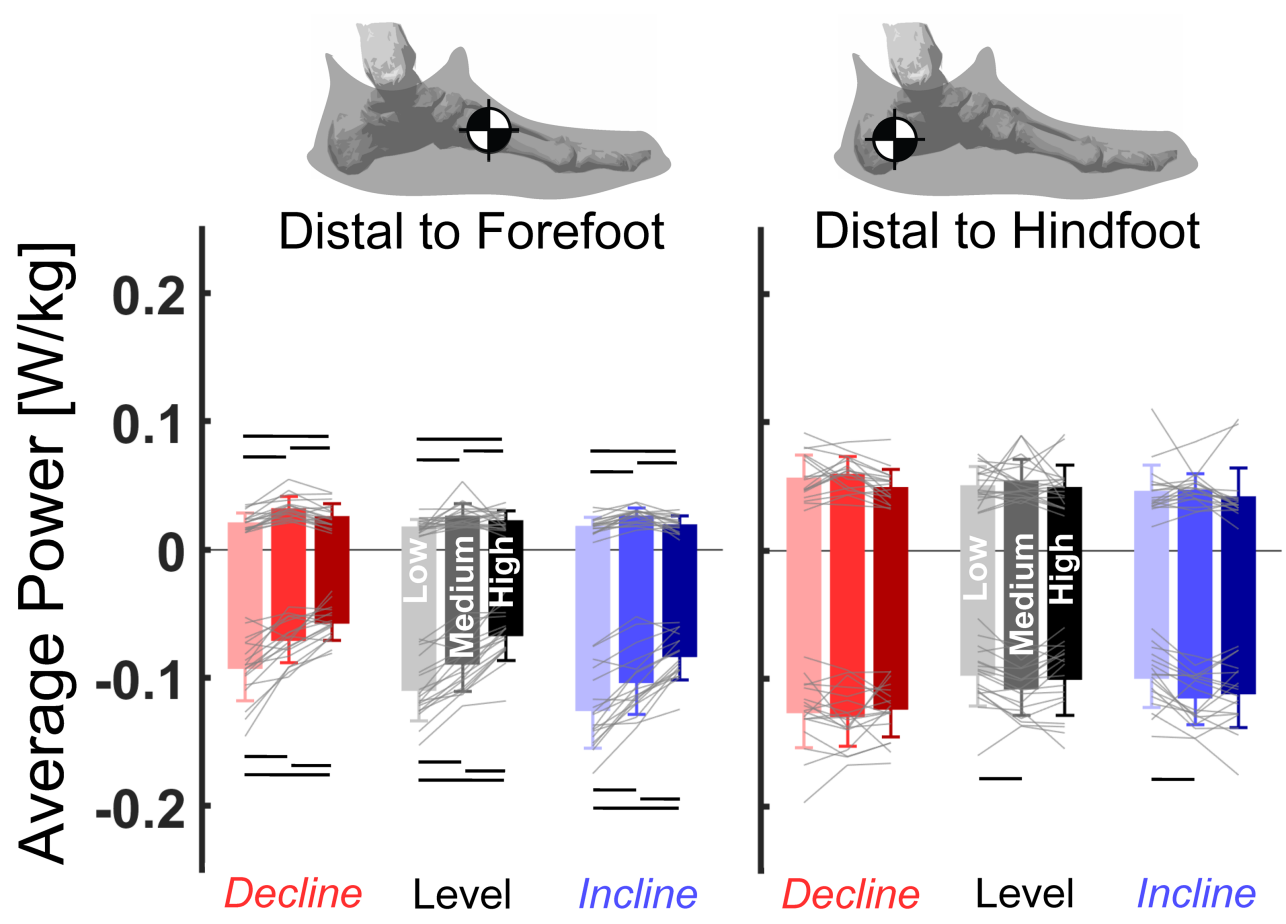
Average positive and negative power (*N*=*18*) of foot+shoe structures. Power distal to the forefoot center of mass (*N* = *18*) is displayed on the left and distal to the hindfoot center of mass (*N* = *18*) is on the right. Plots are grouped by slope condition (red = Decline, grey/black = Level, blue = Incline). Within each slope group, darkening color from left to right represents increasing footwear stiffness. Horizontal lines represent statistically significant pairwise differences between footwear stiffness conditions within each slope condition following the detection of a main stiffness effect or a slope stiffness interaction effect using two-way repeated measures ANOVA (*p* < 0.05). Pairwise differences between slope conditions are detailed in File S1. The reader is directed to File S2 for analysis of average positive and negative midtarsal joint (*i.e.,* arch) power.

The foot+shoe structures distal to the forefoot center of mass performed a smaller magnitude of average negative power with increasing stiffness (F(2,36) = 84.3933, ${\eta }_{p}^{2}=0.76$, *p* < 0.001; Low *vs.* Medium: *d* = 1.43, *p* < 0.001, Low *vs.* High: *d* = 2.29, *p* < 0.001, Medium *vs.* High: *d* = 1.32, *p* < 0.001) (Fig. 2). These structures produced the greatest average positive power in the Medium stiffness condition, followed by the High stiffness condition, then the Low stiffness condition (F(2,36) = 27.2162, ${\eta }_{p}^{2}=0.82$, *p* < 0.001; Low *vs.* Medium: *d* = 1.09, *p* < 0.001, Low *vs. High: d* = 0.57, *p* < 0.001, Medium *vs.* High: *d* = 0.59, *p* < 0.001).

### Foot+Shoe power distal to the hindfoot

There was an interaction between footwear stiffness and walking slope for the average negative Foot+Shoe Power Distal to the Hindfoot (F(4,72) = 2.7815, ${\eta }_{p}^{2}=0.13$, *p* = 0.0325). Follow-up one-way repeated measures ANOVAs and *post-hoc* pairwise comparisons for the effect of footwear stiffness within each walking slope revealed a greater magnitude of average negative power in the Medium compared with the Low stiffness condition during Level (*d* = 0.74, *p* = 0.003) and Incline (*d* = 1.35, *p* = 0.001) walking (Fig. 2). Footwear stiffness did not statistically significantly alter average positive Foot+Shoe Power Distal to the Hindfoot (F(2,36) = 2.7003, ${\eta }_{p}^{2}=0.13$, *p* = 0.0848).

### Ankle joint mechanics

**Figure 3:**
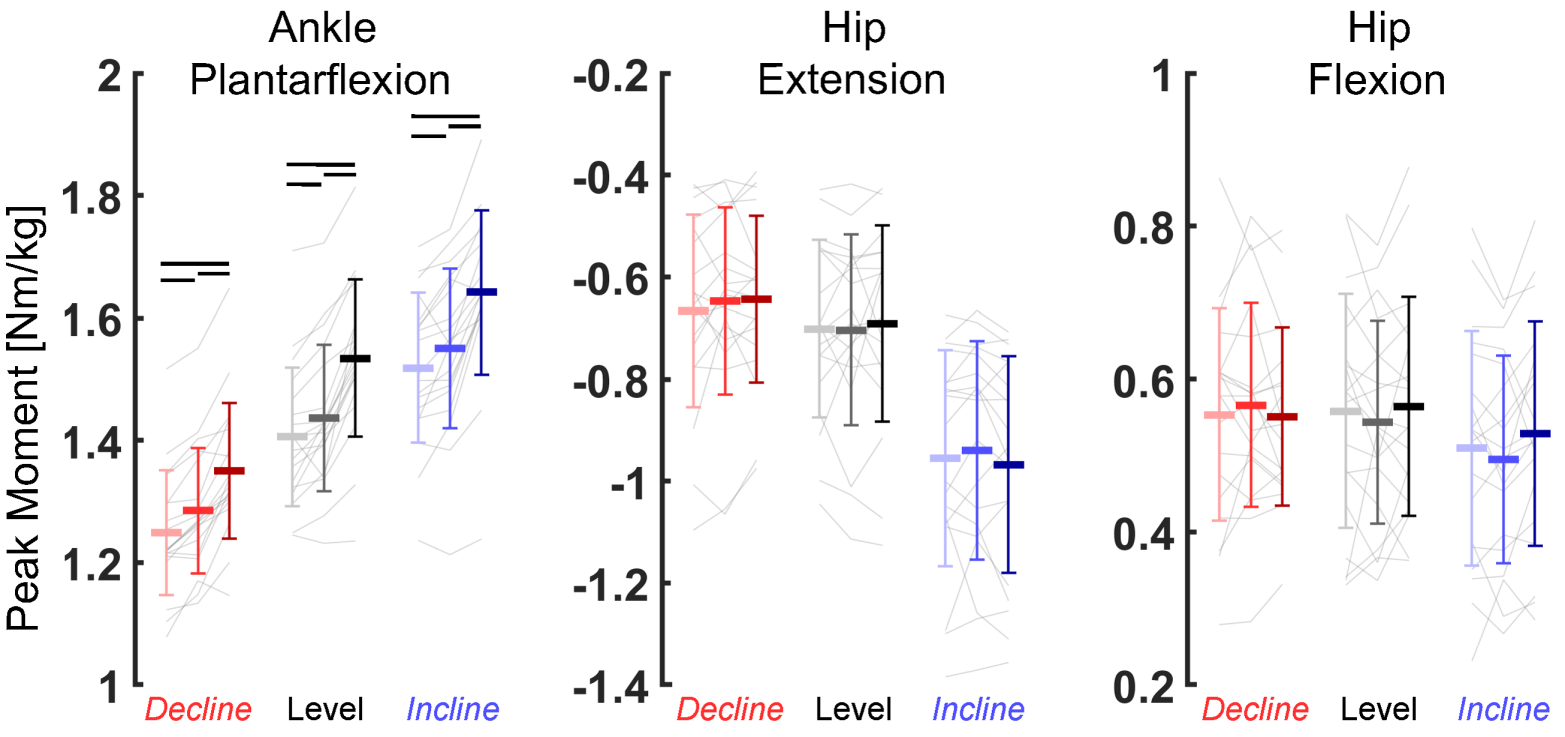
Peak joint moment (*N*=*19*) for ankle plantarflexion (left), hip extension (middle), and hip flexion (right). Plots are grouped by slope condition (red = Decline, grey/black = Level, blue = Incline). Within each slope group, darkening color from left to right represents increasing footwear stiffness. Horizontal lines represent statistically significant pairwise differences between footwear stiffness conditions within each slope condition following the detection of a main stiffness effect or a slope stiffness interaction effect using two-way repeated measures ANOVA (*p* < 0.05). Pairwise differences between slope conditions are detailed in File S1.

**Figure 4:**
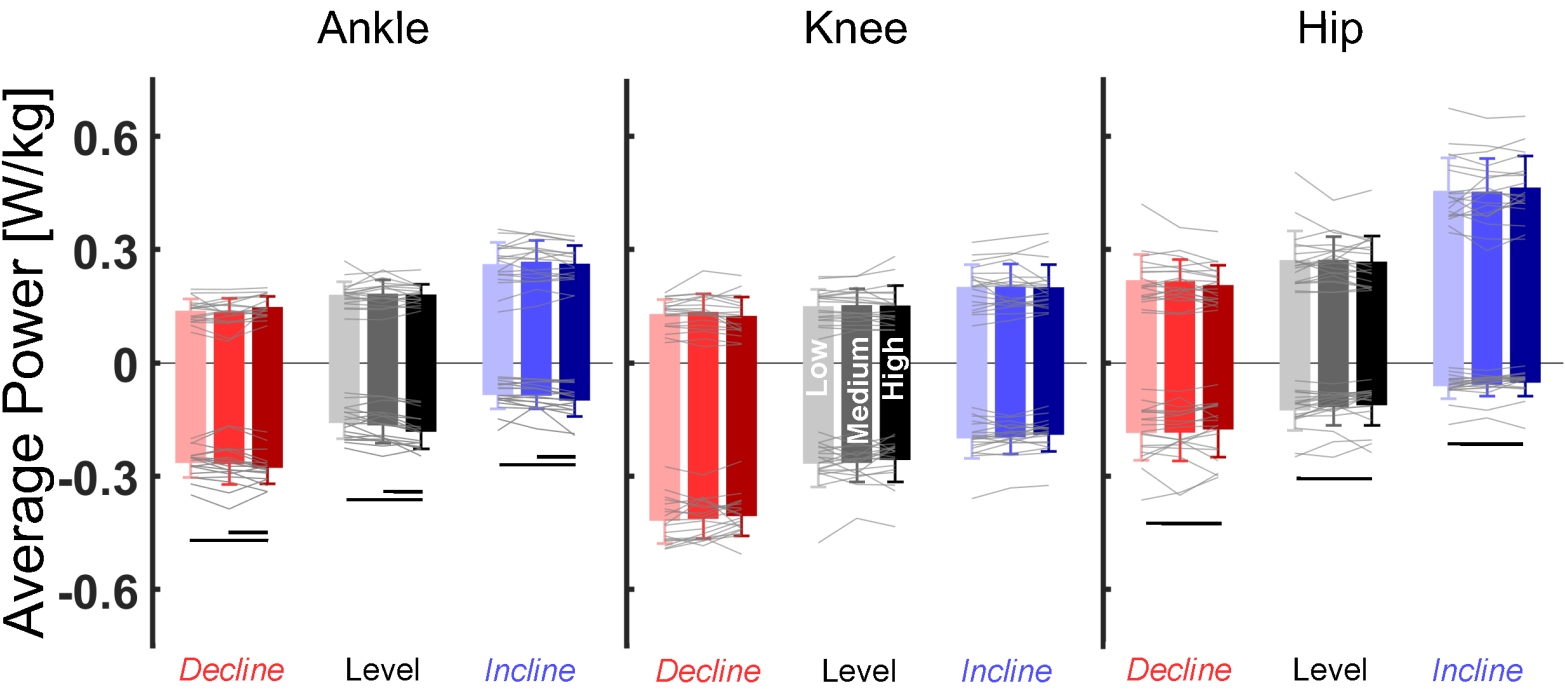
Average positive and negative six degrees of freedom joint power for the ankle (*N*=*18*) (left), knee (*N*=*19*) (center), and hip (*N*=*19*) (right). Plots are grouped by slope condition (red = Decline, grey/black = Level, blue = Incline). Within each slope group, darkening color from left to right represents increasing footwear stiffness. Horizontal lines represent statistically significant pairwise differences between footwear stiffness conditions within each slope condition following the detection of a main stiffness effect or a slope stiffness interaction effect using two-way repeated measures ANOVA (*p* < 0.05). Pairwise differences between slope conditions are detailed in File S1.

With increasing footwear stiffness, the peak ankle moment increased (F(2,38) = 136.5921, ${\eta }_{p}^{2}=0.87$, all pairwise *p* < 0.001; Low *vs.* Medium: *d* = 0.89, *p* < 0.001, Low *vs.* High: *d* = 2.33, *p* < 0.001, Medium *vs.* High: *d* = 1.66, *p* < 0.001) (Fig. 3). Increasing stiffness increased the magnitude of average negative power performed (F(2,36) = 9.8358, ${\eta }_{p}^{2}=0.35$, *p* < 0.001) (Fig. 4). Specifically, average negative power was greater in the High compared with the Low (*d* = 0.82, *p* < 0.001) and Medium stiffness condition (*d* = 0.64, *p* < 0.001) (Fig. 4). Footwear stiffness and walking slope interacted in terms of their effect on average positive ankle joint power (F(4,72) = 2.8286, ${\eta }_{p}^{2}=0.15$, *p* = 0.0341). Follow-up one-way repeated-measures ANOVAs revealed no effect of footwear stiffness at any walking slope (Fig. 4).

### Knee and hip joint mechanics

There was no effect of footwear stiffness on knee joint average negative (F(2,38) = 2.8831, ${\eta }_{p}^{2}=0.14$, *p* = 0.0722) or positive (F(2,38) = 0.7299, ${\eta }_{p}^{2}=0.04$, *p* = 0.4944) power. There was also no effect of footwear stiffness on early stance peak hip extension moment (F(2,38) = 0.2867, ${\eta }_{p}^{2}=0.02$, *p* = 0.7499) (Fig. 3). An interaction effect was present for late stance peak hip flexion moment (F(4,76) = 2.6346, ${\eta }_{p}^{2}=0.12$, *p* = 0.0408) (Fig. 3). However, follow-up one-way repeated-measures ANOVA indicated no effect of footwear stiffness at any walking slope. Increasing footwear stiffness altered the average negative power at the hip (F(2,38) = 3.8028, ${\eta }_{p}^{2}=0.16$, *p* = 0.0326) (Fig. 4). The High stiffness condition resulted in a decreased magnitude of average negative power compared with the Low stiffness condition (*d* = 0.45, *p* = 0.001) (Fig. 4). Follow-up one-way repeated measures ANOVAs conducted due to the stiffness × slope interaction effect on average positive hip joint power (F(4,76) = 2.5525, ${\eta }_{p}^{2}=0.16$, *p* = 0.0457) revealed no effects of stiffness within any slope condition (Fig. 4).

### Metabolic energetics

There was no statistically significant main effect of footwear stiffness (F(2,38) = 0.4888, ${\eta }_{p}^{2}=0.02$, *p* = 0.6257) nor a slope × stiffness interaction effect (F(4,76) = 0.8457, ${\eta }_{p}^{2}=0.06$ *p* = 0.4955) on net metabolic power.

## Discussion

The purpose of this study was to assess how footwear stiffness influences older adult mechanical and metabolic energetics across everyday walking slopes. We hypothesized that footwear stiffening *via* carbon fiber insoles would reduce older adult metabolic costs by targeting specific age-related ankle and foot mechanical deficits. Here, we demonstrated that stiffening older adults’ footwear does not reduce energy lost by the entire foot+shoe complex (*i.e.,* Foot+Shoe Power Distal to the Hindfoot) but does increase peak ankle joint moments. Increasing footwear stiffness did not result in changes to the mechanics of the knee and hip joints, nor a consistent reduction in older adults’ metabolic cost. This is important information for future interventions hoping to target increased older adult metabolic costs *via* footwear intervention.

### Energetics of the foot+shoe complex

With increased footwear stiffness, we found unique mechanical effects at different regions of the foot. When considering the Foot+Shoe Power Distal to the Forefoot (*i.e.,* energy fluctuations of foot and shoe structures distal to the forefoot center of mass), we found a reduction in energy loss which is in line with previous analyses (*e.g.*, Hoogkamer, Kipp & Kram, 2019; Takahashi et al., 2016; Stefanyshyn & Nigg, 2000). However, the Foot+Shoe Power Distal to the Hindfoot (*i.e.,* energy fluctuations of the entire foot+shoe complex) did not demonstrate a similar reduction. Instead, average negative power slightly increased in magnitude with stiffer footwear (Fig. 2). These findings highlight the energetic interplay within the foot (discussed in detail in the following paragraph) that should be considered in footwear analyses and interventions. If reducing energy loss from the entire foot+shoe complex is the goal, these results suggest that more complex insole designs or other factors beyond increasing footwear stiffness may need to be explored.

We posit that the region-specific effects of increased footwear stiffness on foot energetics reflect the coordinated function of the foot’s metatarsophalangeal joint and the foot’s arch. In barefoot walking, structures crossing the metatarsophalangeal joint appear to be absorbing mechanical energy late in stance at the same time as those crossing the arch appear to be generating mechanical energy (for example, see MacWilliams, Cowley & Nicholson, 2003). Many biological structures cross both the metatarsophalangeal joints and the arch, leading multiple authors to postulate that these distinct energetics arise due to energy transfer within the foot (Davis & Challis, 2023; McDonald et al., 2016; Welte et al., 2023; Williams, Arch & Bruening, 2023). Under this energy transfer paradigm, reducing the energy absorbed about the metatarsophalangeal joint with increasing footwear stiffness would reduce the positive mechanical energy about the arch. We performed a secondary analysis of midtarsal joint (*i.e.,* arch) six degrees of freedom mechanical power to investigate this phenomenon. In line with the energy transfer paradigm, our results indicate that the arch is producing less mechanical energy with increasing footwear stiffness (File S2). If energy transfer within the foot explains the present results, reductions in both positive arch power and negative Foot+Shoe Power Distal to the Forefoot should mostly offset one another. The energetics of the entire foot and shoe (*i.e.,* Foot+Shoe Power Distal to the Hindfoot) would then be expected to remain fairly similar with increasing footwear stiffness. The Foot+Shoe Power Distal to the Hindfoot results demonstrate that this is the case (Fig. 2). These findings provide further evidence of within foot energy transfer, and that attenuating negative power at the distal portion of the foot similarly attenuates positive power produced about the foot’s arch during propulsion.

### Lower-limb joint mechanics and metabolic power

**Figure 5:**
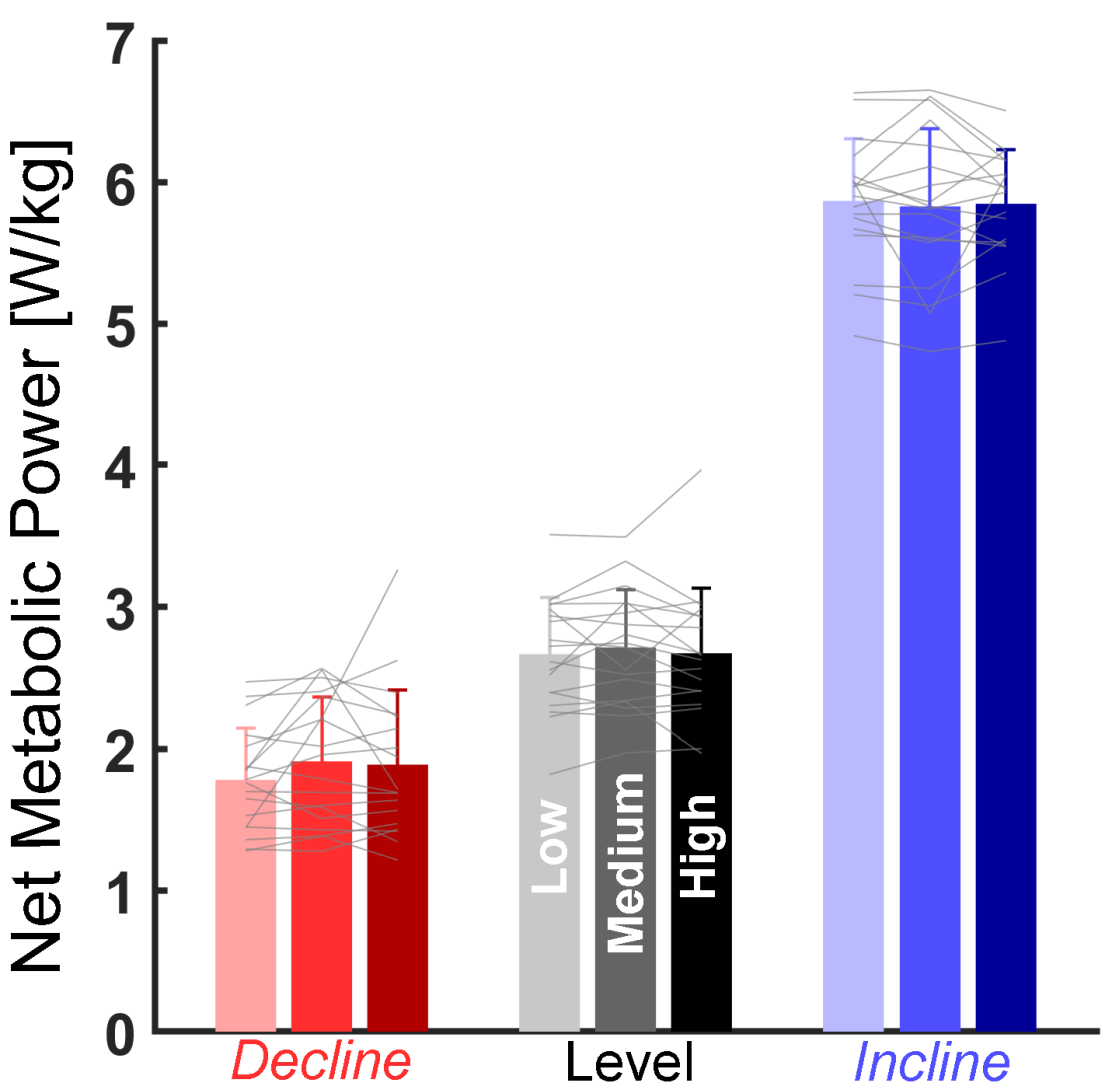
Net metabolic power (N = 18) during walking. Plots are grouped by slope condition (red = Decline, grey/black = Level, blue = Incline). Within each slope group, darkening color from left to right represents increasing footwear stiffness. Pairwise differences between slope conditions are detailed in File S1.

We hypothesized that reducing a well-known age-related impairment—reduced ankle joint moment generation—would result in alterations to proximal joint mechanics that coincided with reductions in metabolic cost. Across slopes, our older adults walked with ∼10% greater peak ankle joint moments in the High compared with the Low stiffness conditions (Fig. 3). Similar benefits to ankle joint moment have been demonstrated in older adults after 12 sessions of walking against horizontal impeding forces (Conway et al., 2021). These increases to peak ankle joint moment fall within the range of reported older adult ankle joint moment deficits (5% (Buddhadev, Smiley & Martin, 2020) to 25% (DeVita & Hortobagyi, 2000)). However, despite greater peak ankle joint moments in the present study, neither older adult hip and knee joint mechanics nor their net metabolic power were statistically significantly different at the group level (Figs. 3–5). Our findings suggest that targeting older adults’ ankle joint moment deficits *via* footwear stiffening falls short of attenuating older adults’ distal to proximal shift in joint mechanics or reducing their metabolic cost of walking. This may indicate that deficits in ankle joint moment are not a dominant contributor to older adult’s metabolic inefficiency in general. However, it is also possible that the present results are specific to increases in ankle joint moment brought about by increased footwear longitudinal bending stiffness. The present results cannot distinguish between these possibilities, and therefore the ankle joint’s contribution to older adult gait deficits remains an important question for future analysis.

Consistent hip and knee joint mechanics in the presence of altered foot and ankle mechanics have been demonstrated in previous footwear investigations. For example, younger adults walking with these same carbon fiber insoles demonstrate greater peak ankle dorsiflexion and reduced peak ankle plantarflexion angles but no such differences at the knee or hip (Ray & Takahashi, 2020). Individuals similarly altered their ankle joint mechanics but maintained more consistent knee, hip, and pelvis kinematics across a range of shoe rocker profiles (Wang & Hansen, 2010). Modern advanced running footwear technology –wherein both carbon fiber plates and altered shoe rocker profiles are used –appear to alter foot and ankle joint mechanics without substantially perturbing hip and knee joint mechanics as well (Hoogkamer, Kipp & Kram, 2019; Hunter et al., 2019). Despite similar proximal joint mechanics, these studies have demonstrated that shoe properties can influence the metabolic cost of walking and running. This would suggest that it is not the lack of effect on knee and hip mechanics in the present study that led to a lack of effect on metabolic rate. Rather, the specific foot and ankle effects from flat carbon fiber insoles used here are not metabolically advantageous at the whole-body level. Future investigations of muscle-level effects across the lower-limb joints are needed to examine how distal and proximal joint mechanics may be interacting to bring about metabolic benefits and/or penalties in footwear with differing properties.

## Limitations

The present results must be considered in light of limitations. First, the older adults who participated in this study were free of neurological, musculoskeletal, and metabolic complications that substantially influenced their walking ability. Among the older adult population, these complications are widespread (US Department of Health and Human Services, 2021). As a result, the present findings may be restricted to relatively high-functioning older adults.

Additionally, we saw substantial individual variability in our participant metabolic responses to increasing footwear stiffness that may be attributed to study limitations. We opted not to counterbalance (*e.g.*, ABCCBA) the footwear condition order, as is becoming more common in footwear research (Healey & Hoogkamer, 2022; Hoogkamer et al., 2018; Hunter et al., 2019) to reduce participant burden across the nine experimental conditions. Instead, we randomized the order participants completed each shoe condition, which served to limit any potential ordering effects at the group level. While we allowed our participants to take more than the recommended number of steps to acclimate to new athletic footwear (Soares et al., 2024), our results may have differed if more time was allowed for exploration, and it may be that certain participants “figured out” how to walk more efficiently in the different footwear conditions. It is also possible that baseline characteristics (*e.g.*, sex, height, body-mass) interacted with the effects of the footwear condition. Examining these factors was outside the scope of the current work, but future analyses powered to detect these relationships should investigate factors that delineate those who benefit from those who do not, and how training could influence the ability to achieve benefits.

It should also be noted that collecting mechanical and metabolic data on separate days may weaken potential associations between them. Changes in foot mechanical energetics and peak ankle joint moments with increasing footwear stiffness were consistent across participants, suggesting that these changes would not wash out due to between-day variability. Knee and hip joint kinetic changes, however, were more variable between footwear conditions and have been previously demonstrated to be more variable between testing sessions (intersession intraclass correlation coefficients of between 0.48–0.89; (Wilken et al., 2012)). Despite this intersession variability, metabolic variables demonstrate strong intersession intraclass correlation coefficients (≥0.81) in both younger and older adults (Darter, Rodriguez & Wilken, 2013; Wert et al., 2015). As such, we do not attribute the lack of consistent effects of footwear stiffness on participants’ metabolic power to between-day variability in lower-limb joint mechanics.

## Conclusions

The present findings indicate that increasing older adult footwear bending stiffness (1) alters the energetics of the foot’s structures in an interdependent manner: decreasing the negative Foot+Shoe Power Distal to the Forefoot attenuates positive power performed by more proximal structures in the foot (*e.g.*, the foot’s arch); (2) increases peak ankle joint moments without altering ankle, knee, or hip joint power production or hip joint moment; and (3) does not lead to consistent changes in the metabolic cost of walking. The effects of stiffer footwear on other aspects of gait besides metabolic economy that are no less important to older adults (*e.g.*, walking speed, balance) are attractive avenues for future study with the goal of improving older adult mobility.

## Supplemental Information

10.7717/peerj.21563/supp-1 Supplemental Information 1 Statistical effects of walking slope, i.e., main effects and *post-hoc* pairwise comparisons between slope conditions

10.7717/peerj.21563/supp-2 Supplemental Information 2 Six degrees of freedom midtarsal joint power (*N = 18*) across slopes and footwear stiffness conditions

## Additional Information and Declarations

### Data Availability

The following information was supplied regarding data availability: The data is available at Zenodo: - Davis, D. (2026). Data for Davis et al. ”Can increasing footwear bending stiffness ameliorate age-related mechanical and metabolic deficits in walking?” [Data set]. Zenodo. https://doi.org/10.5281/zenodo.18088742.
