# Supplementary material for: Can increasing footwear bending stiffness ameliorate age-related mechanical and metabolic deficits in walking?
Source: PeerJ. 2026 Jul 30;14:e21563. doi: 10.7717/peerj.21563 (PMC13429106; doi:10.7717/peerj.21563)
Supplement: Supplemental Information 1 [file peerj-14-21563-s001.docx]

# **APPENDIX A**

## *Slope Effects*

Statistically significant main effects of slope only (i.e., no main effect of stiffness or a slope $\times$ stiffness interaction) on lower-limb mechanics and net metabolic power are presented in this appendix. References are made to figures in the main manuscript text.

### *Distal to Forefoot Mechanics*

Distal to forefoot average negative power increased in magnitude from Decline, to Level, to Incline walking (F(2,36) = 56.9586, p < 0.001). The average positive power of structures distal to the forefoot decreased (F(2,36) = 5.4685, p = 0.011), specifically between the Decline and Level condition and the Decline and Incline condition (both p < 0.001).

### *Distal to Hindfoot Mechanics*

In the Low stiffness condition, the magnitude of average negative power was greater in the Decline condition than the other two slopes (both p < 0.001) (Main Text Figure 2). In the Medium stiffness condition, all slope conditions were statistically significantly different (all p < 0.0288). Lastly, in the high stiffness condition, the Level condition was statistically significantly different than both the Incline (p = 0.0286) and Decline conditions (p = 0.0112) (Main Text Figure 2).

### *Ankle Joint Mechanics*

Average positive ankle joint power increased from the Decline, to Level, to Incline conditions, and all pairwise comparisons of slope within a given stiffness condition were statistically significant (p < 0.001), with (Main Text Figure 4). The magnitude of peak ankle joint moment (F(2,38) = 349.8547, p < 0.001) was greatest in the Incline walking condition, followed by Level walking, and was the smallest in Decline walking (all p < 0.001) (Main Text Figure 3). From Decline, to Level, to Incline walking, the ankle joint average negative power decreased in magnitude (F(2,36) = 174.7241, p < 0.001; all post-hoc comparisons p < 0.001). Follow-up pairwise comparisons of average positive power within each footwear stiffness condition revealed that all walking slopes were statistically significantly different (all p < 0.001), with power increasing from the Decline, to Level, to Incline condition (Main Text Figure 4).

### *Knee and Hip Joint Mechanics*

The magnitude of average negative knee joint power was greatest during Decline walking, followed by Level, and was smallest in Incline walking (F(2,38) = 182.4122, p < 0.001, all pairwise comparisons p < 0.001) (Main Text Figure 4). The opposite was true for average positive knee joint power, which increased from Decline, to Level, to Incline walking (F(2,38) = 65.6442, p < 0.001, all pairwise comparisons p < 0.001) (Main Text Figure 4).

Peak hip joint extension moment was sensitive to walking slope (F(2,38) = 100.385, p < 0.001), increasing with slope (Main Text Figure 3). Pairwise comparisons of peak hip flexion moment within each footwear condition revealed statistically significant differences between the Level and Incline walking conditions in the Low (p = 0.0036) and Medium (p < 0.001) footwear stiffness conditions, and a difference between the Decline and Incline walking conditions in the Medium footwear stiffness condition (p < 0.001) (Main Text Figure 3). Lastly, the magnitude of average negative power decreased from Decline to Level (p < 0.001) and positive power increased from Level to Incline conditions (Main Text Figure 4).

### *Net Metabolic Power*

Net metabolic power was sensitive to walking slope (F(2,38) = 2,057.9, p < 0.001), increasing from Decline, to Level, to Incline walking (all p < 0.001) (Main Text Figure 5).
