# Supplementary material for: Can increasing footwear bending stiffness ameliorate age-related mechanical and metabolic deficits in walking?
Source: PeerJ. 2026 Jul 30;14:e21563. doi: 10.7717/peerj.21563 (PMC13429106; doi:10.7717/peerj.21563)
Supplement: Supplemental Information 2 [file peerj-14-21563-s002.docx]

We computed six degrees of freedom midtarsal joint power using the foot model of Bruening et al. (2012). Power time series were then time-integrated then divided by stride time and participant body mass to compute average positive and negative power [W/kg].


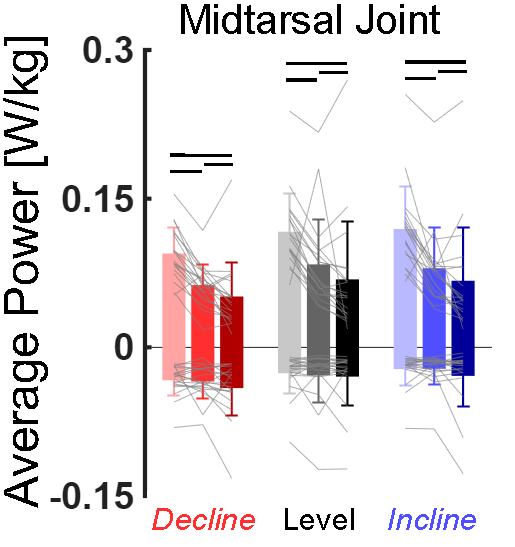


Average positive and negative six degrees of freedom midtarsal joint (i.e., arch) power (*N = 18*). Plots are grouped by slope condition (red = Decline, grey/black = Level, blue = Incline). Within each slope group, darkening color from left to right represents increasing footwear stiffness. Horizontal lines represent statistically significant pairwise differences between footwear stiffness conditions within each slope condition following the detection of a main stiffness effect or a slope stiffness interaction effect using two-way repeated measures ANOVA (α < 0.05).

There were main effects of both slope (F(2,36) = 11.6712, $\eta_{p}^{2}$ = 0.39, p < 0.001) and stiffness (F(2,36) = 43.3635, $\eta_{p}^{2}$ = 0.71, p < 0.001) on average positive midtarsal joint power. With increasing slope (from decline to level to incline), positive power generally increased (*Decline* vs. *Level: d* = 0.70, p < 0.001*, Decline* vs. *Incline: d* = 0.72, p < 0.001). With increasing stiffness, average positive power decreased (*Low* vs. *Medium: d* = 1.18, p < 0.001*, Low* vs. *High: d* = 2.95, p < 0.001*, Medium vs. High: d* = 0.53, p < 0.001).

Slope also altered the average negative midtarsal joint power (F(2,36) = 26.1862, $\eta_{p}^{2}$ = 0.59, p < 0.001). With increasing slope (from decline to level to incline), negative power generally decreased (*Decline* vs. *Level: d* = 0.83, p < 0.001*, Decline* vs. *Incline: d* = 1.05, p < 0.001, *Level* vs. *Incline: d* = 0.39, p = 0.005).

*References:*

Bruening, D.A., Cooney, K.M., Buczek, F.L., 2012. Analysis of a kinetic multi-segment foot model. Part I: Model repeatability and kinematic validity. Gait Posture 35, 529–534. https://doi.org/10.1016/j.gaitpost.2011.10.363
